# Protein Kinase A Activation Enhances β-Catenin Transcriptional Activity through Nuclear Localization to PML Bodies

**DOI:** 10.1371/journal.pone.0109523

**Published:** 2014-10-09

**Authors:** Mei Zhang, Emilia Mahoney, Tao Zuo, Parmeet K. Manchanda, Ramana V. Davuluri, Lawrence S. Kirschner

**Affiliations:** 1 Department of Molecular, Virology, Immunology, and Medical Genetics, The Ohio State University, Columbus, Ohio, United States of America; 2 Division of Endocrinology, Diabetes and Metabolism, The Ohio State University, Columbus, Ohio, United States of America; Western University, Canada

## Abstract

The Protein Kinase A (PKA) and Wnt signaling cascades are fundamental pathways involved in cellular development and maintenance. In the osteoblast lineage, these pathways have been demonstrated functionally to be essential for the production of mineralized bone. Evidence for PKA-Wnt crosstalk has been reported both during tumorigenesis and during organogenesis, and the nature of the interaction is thought to rely on tissue and cell context. In this manuscript, we analyzed bone tumors arising from mice with activated PKA caused by mutation of the PKA regulatory subunit Prkar1a. In primary cells from these tumors, we observed relocalization of β-catenin to intranuclear punctuate structures, which were identified as PML bodies. Cellular redistribution of β-catenin could be recapitulated by pharmacologic activation of PKA. Using 3T3-E1 pre-osteoblasts as a model system, we found that PKA phosphorylation sites on β-catenin were required for nuclear re-localization. Further, β-catenin's transport to the nucleus was accompanied by an increase in canonical Wnt-dependent transcription, which also required the PKA sites. PKA-Wnt crosstalk in the cells was bi-directional, including enhanced interactions between β-catenin and the cAMP-responsive element binding protein (CREB) and transcriptional crosstalk between the Wnt and PKA signaling pathways. Increases in canonical Wnt/β-catenin signaling were associated with a decrease in the activity of the non-canonical Wnt/Ror2 pathway, which has been shown to antagonize canonical Wnt signaling. Taken together, this study provides a new understanding of the complex regulation of the subcellular distribution of β-catenin and its differential protein-protein interaction that can be modulated by PKA signaling.

## Introduction

Both the protein kinase A (PKA) and Wnt signaling pathways play important fundamental roles in most tissue types. These signaling cascades play complex roles in differentiation, development, and tumorigenesis, where effects within a specific tissue or developmental stage are highly dependent on context. Proper regulation of these pathways is required for normal formation of both epithelial and mesenchymal tissue, such as bone and muscle [1], [2], [3], [4].

The protein kinase A (PKA) pathway has been known to be involved in bone biology since the elucidation of the role of parathyroid hormone (PTH) in bone homeostasis. PTH signals through its 7 transmembrane G protein coupled receptor PTH Receptor 1 (PTHR1) to activate the PKA signaling cascade [5], modeling of which has been shown to be required for proper skeletal formation and function in mammals. Regulation of the pathway is complex, as intermittent stimulation of PKA signaling by physiologic or intermittent dosing of parathyroid hormone (or its synthetic congener teriparatide, Forteo) promotes bone accumulation, whereas extended exposure promotes bone resorption, as in primary hyperparathyroidism.

There are also a number of human diseases of bone which are directly related to dysregulated of the PKA signaling pathway. Hyperactivation of the PKA pathway can cause proliferation of undermineralized bone, as can be observed in McCune-Albright Syndrome (MAS, OMIM 174800) and Carney Complex (CNC, OMIM 160980). The former is caused by activating mutations of the stimulatory G protein Gsα, leading to constitutive PKA excess driven through dysregulation of cAMP generation by the mutant G protein [6]. In CNC, inactivating mutations in *PRKAR1A*, the type 1A regulatory subunit, cause hyperactivation of PKA directly at the level of the PKA holoenzyme [7]. Although histologically distinguishable, the bone pathology of these two human syndromes is quite similar as would be expected from the similarity of their biochemistry [8]. The undermineralization of bone caused by PKA activation in these syndromes has also been modeled *in vitro* using introduction of activated GNAS1 or shRNA-mediated knockdown of Prkar1a [9], [10]. Conversely, under activity of the PKA pathway causes excessive bone deposition as observed in Progressive Osseous Heteroplasia (OMIM 166350) or the aberrant bone deposition in Albright's Hereditary Osteodystrophy (OMIM 103580/612463)

β-catenin is a multifunctional protein that serves as a component of the cell-cell adherens junctions as well as a transcriptional regulator of the canonical Wnt signaling pathway [11]. In the latter role, β-catenin transcriptionally activates growth-related genes, such as cyclin D1, through collaboration with T-cell factor (TCF)/lymphoid enhancer factor (LEF) transcription factors [12]. β-catenin activity is generally controlled by regulating its abundance through a series of N-terminal phosphorylation events carried out by Casein Kinase I (CK1) and glycogen synthase kinase-3 (GSK3β) [13], [14]. Phosphorylation of β-catenin by these kinases leads to degradation triggered by the Axin destruction complex. Physiologically, it has been shown that the Wnt/β-catenin pathway plays a critical role in regulating osteoblast development and differentiation. Specifically, activation of Wnt/β-catenin pathway in progenitor cells can also lead to an arrest of osteoblast differentiation [15], [16]. Like the PKA pathway, alterations in Wnt signaling have been found to cause human bone disease. Mutations in the Wnt co-receptor LRP5 can be associated with low (Osteoporosis- Pseudoglioma Syndrome; OPPG OMIM 259770) or high bone mass, depending on whether the mutation is inactivating or activating, respectively [17]. Human mutations in the Wnt antagonist Sclerostin (SOST) also cause high bone mass through loss of pathway inhibition (Van Buchem disease OMIM 239100 or Sclerostosis OMIM 269500) [18].

Crosstalk between these two pathways has previously been demonstrated by the fact that PKA has been shown to phosphorylate β-catenin in its C-terminus at serines 552 and 675, although the effects of this post-translational modification is unclear [19], [20]. Activation of PKA was also felt to promote Wnt signaling in a subset of adrenal tumors and cancers [21]. Conversely, Wnt signaling during developing myoblasts required CREB and PKA activity, so cross-talk appears to go both ways [22].

In this report, we describe alterations in β-catenin that are observed in bone tumors arising from mice with mutations in *Prkar1a* as a means to activate PKA signaling [23]. Investigation of this phenomenon led us to reassess the interaction of PKA and Wnt/β-catenin pathways in the osteoblastic cell lineage and explore the mechanisms by which PKA regulates Wnt/β-catenin signaling. We report that PKA activation leads to nuclear relocalization of β-catenin to PML bodies, and that this process requires PKA-mediated phosphorylation. These studies point to more complex regulation of Wnt signaling, and how this pathway may be modulated by PKA signaling.

## Materials and Methods

All research performed using animals was carried out in accordance with the highest standards of animal care under research protocols (2002-A-0097 and 2008-A-0104) approved by The Ohio State University Institutional Animal Care and Use Committee (IACUC).

### Cell culture and generation of stable knockdown cell lines

Primary cultures of mouse tumors and normal bones were prepared and grown in Dulbecco's Modified Eagle Medium (DMEM) supplemented with 10% fetal bovine serum (Hyclone, Logan, UT),10,000 U/liter penicillin G, 10 mg/liter streptomycin, and 1% ascorbic acid (osteoblast medium). as described previously [23]. The murine pre-osteoblast cell line, MC3T3-E1, subclone 4 (CRL-2593, ATCC) was cultured in alpha modified Minimum Essential Medium (MEM α) supplemented with 10% FBS, and antibiotics.

Stable Prkar1a knockdown was achieved as previously described [24]. Briefly, lentiviral vectors with LKO.1 backbone expressing shRNA for mouse Prkar1a (Open Biosystems, clone ID: TRCN0000025488) or lentiviral LKO.1 empty vector control recommended by the RNAi Consortium (Open Biosystems) were generated by cotransfection of 293T cells with the packaging plasmids. Viruses were collected 48 h after transfection, filtered with a 0.45-µm filter, and used to infect MC3T3-E1 cells. The transduced cells were selected with 5 µg/mL puromycin.

### Transient transfection and Luciferase assay

FLAG-tagged β-catenin was obtained from Addgene (Plasmid 16828, Cambridge, MA) and the β-catenin mutants were generated using Site-Directed Mutagenesis (Stratagene, La Jolla, CA). All mutants were sequence verified prior to use. The CFP-PML expression plasmid [25] was the gracious gift of Dr. Marc Tini (University of Western Ontario). Transfection was performed using Xtreme HD transfection reagent (Roche) according to the manufacturer's instructions followed by the treatment with vehicle [dimethylsulfoxide (DMSO)] or forskolin (50 µM).

To measure the Wnt/β-catenin pathway mediated TCF/LEF-1 transcriptional activity, MC3T3-E1 cells were transfected with TCF-luciferase reporter (TOPFlash) or its mutated control reporter (FOPFlash) along with WT or mutants of β-catenin. The luciferase activity was measured using the dual-luciferase assay kit (Promega).

### Immunofluorescence and confocal microscopy

Cells were cultured on coverslips, fixed with 4% paraformaldehyde in PBS for 10 min and permeabilized with 0.1% Triton X-100 in PBS for 5 min. Coverslips were incubated with primary antibodies for 2–24 h, washed three times with PBS, and incubated with secondary antibodies for 60 min. Samples were mounted using antifade reagent with or without DAPI (ProLong Gold; Invitrogen, Grand Island, NY) and observed using single-photon Olympus FV1000 confocal microscope. To detect the proteins of interest, we used the following antibodies: phosphoSer133-Creb (pS133-Creb), Creb, pS675-β-catenin, pS552-β-catenin, β-catenin and FLAG were from Cell Signaling Technology; PML was from Millipore; Alexa Fluor conjugated secondary antibodies were from Invitrogen.

### RNA isolation and quantitative reverse transcription polymerase chain reaction (QPCR)

RNA was isolated from cells using Trizol and RNeasy Mini Columns (Qiagen, Valencia, CA, USA) and converted to cDNA with the Bio-Rad iScript cDNA SynthesisKit (Bio-Rad Laboratories) according to manufacturer instructions. cDNA was subject to qRT-PCR using the iQ SYBR Green Supermix Kit (Bio-Rad Laboratories) as per the manufacturer's instructions. Reactions were each performed in triplicate. Primers sequences used in this study can be found in Table S1.

### Protein analysis by Western Blot

Whole-cell lysates were prepared using M-PER protein extraction reagent containing HALT protease inhibitors (Pierce, Rockford, IL). For nuclear-cytoplasmic fractionation, cells were lysed in NE-PER extraction reagent (Pierce) and processed according to the manufacturer's instructions. Proteins were resolved by SDS-PAGE gels and transferred to nitrocellulose (Pall, East Hills, NY). Blots were developed with chemiluminescence solution (Western Lightning; PerkinElmer, Norwalk, CT). To detect the proteins of interest, we used the same antibodies described above, with Lamin A and α tubulin antibodies obtained from Santa Cruz Biotechnology (Santa Cruz, CA) and anti-actin from Sigma.

### Statistical Analyses

All experiments were repeated at least three times, and all analyses in this manuscript were performed using a two-sided Student's t test, as implemented by StatCrunch (http://www.statcrunch.com). Differences with p<0.05 were considered significant. All values are expressed as the mean ±S.D. of triplicate independent samples.

## Results

### β-catenin translocates into the nucleus in primary osteoblast cultures when PKA is activated

**Figure 1:**
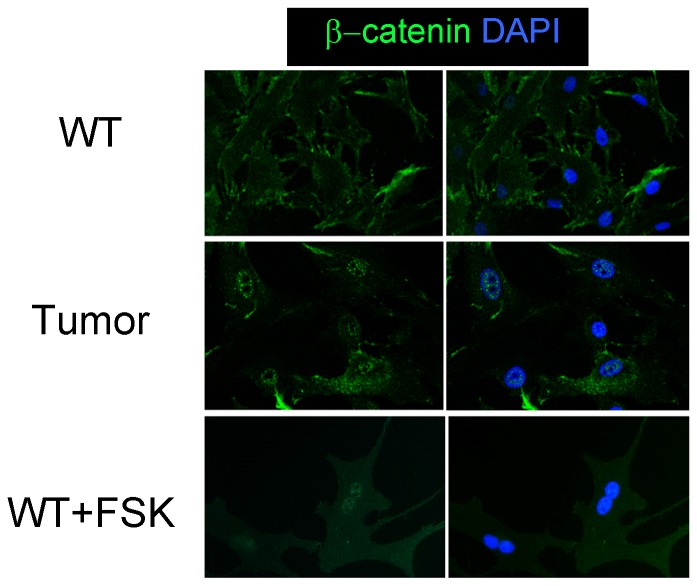
β-catenin forms punctate nuclear lesions in response to PKA activation in primary cultures. Primary osteoblasts from wild type (WT) bones or from bone tumors arising in *Prkar1a^+/−^* mice were studied by immunofluorescence for β-catenin (green). For reference, cell nuclei were stained with DAPI. The left column shows β-catenin, only, whereas the right column shows merger of the β-catenin and DAPI stains. Top) WT osteoblasts. Middle) Tumor osteoblasts. Bottom) WT osteoblasts treated with forskolin (FSK). Note the punctate nuclear localization of β-catenin observed in Tumor cells or WT cells treated with FSK. Magnification: 400x.

We previously reported that approximately 80% of *Prkar1a^+/−^* mice develop osteoblastic bone tumors by one year of age and primary cultures of tumoral bones showed increased PKA activity and decreased osteoblastic differentiation compared to cells isolated from control animals [23]. Because of the known role of β-catenin in bone physiology, we decided to study its role in promoting this bone tumor phenotype. Initial analysis of mRNA and protein expression level of total β-catenin or the GSK-3-phosphorylated form detected no significant differences between osteoblasts from *Prkar1a^+/−^* tumors or control bone (data not shown). However, analysis of β-catenin localization by immunofluorescence (IF) revealed that β-catenin had undergone an alteration of its subcellular distribution in tumor cells (Figure 1). In normal osteoblasts, β-catenin was found at the membrane or was diffusely spread throughout the cell. By contrast, tumor osteoblasts consistently demonstrated increased nuclear localization of β-catenin, with a punctate intranuclear staining pattern. To verify that the intranuclear relocalization was an effect of PKA activation, WT cells were treated with forskolin (FSK) to enable cAMP generation and activation of PKA. Under these conditions, we observed the same pattern of nuclear β-catenin staining as in the tumors (Fig 1, bottom). These data suggest that this particular nuclear distribution of β-catenin results from an increase in PKA activity.

### PKA activation induces β-catenin localization to PML bodies

The appearance of a specific punctate pattern for nuclear β-catenin under these conditions suggested that the protein localized to a specific subnuclear complex or compartment. As proteins that produce the pattern observed for β-catenin are relatively few, we performed co-localization studies for specific proteins, including PML (multifunctional complex), H2AX (DNA double strand breaks), and PCNA (DNA replication). PML is a scaffold protein forming multiprotein complexes which are thought to represent dynamic structures with many intranuclear functions, including transcriptional activation and repression, protein post-translational modification, and protein storage [26]. Previous studies have detected that PML interacts with β-catenin in colorectal cancer cells [27], although little is known about its role in bone. One report [28] suggests that PML overexpression may promote OBL differentiation from mesenchymal stem cells. H2AX is a histone isoform that marks double strand breaks (DSBs) such that H2AX foci are found at the end of DSBs, often induced by radiation. H2AX and β-catenin are not known to interact, although catenin levels may go up in response to DNA damage, and catenins may indirectly influence H2AX levels [29]. Similarly, although β-catenin and PCNA may both be upregulated in proliferating cells, their direct interaction has not been described.

**Figure 2:**
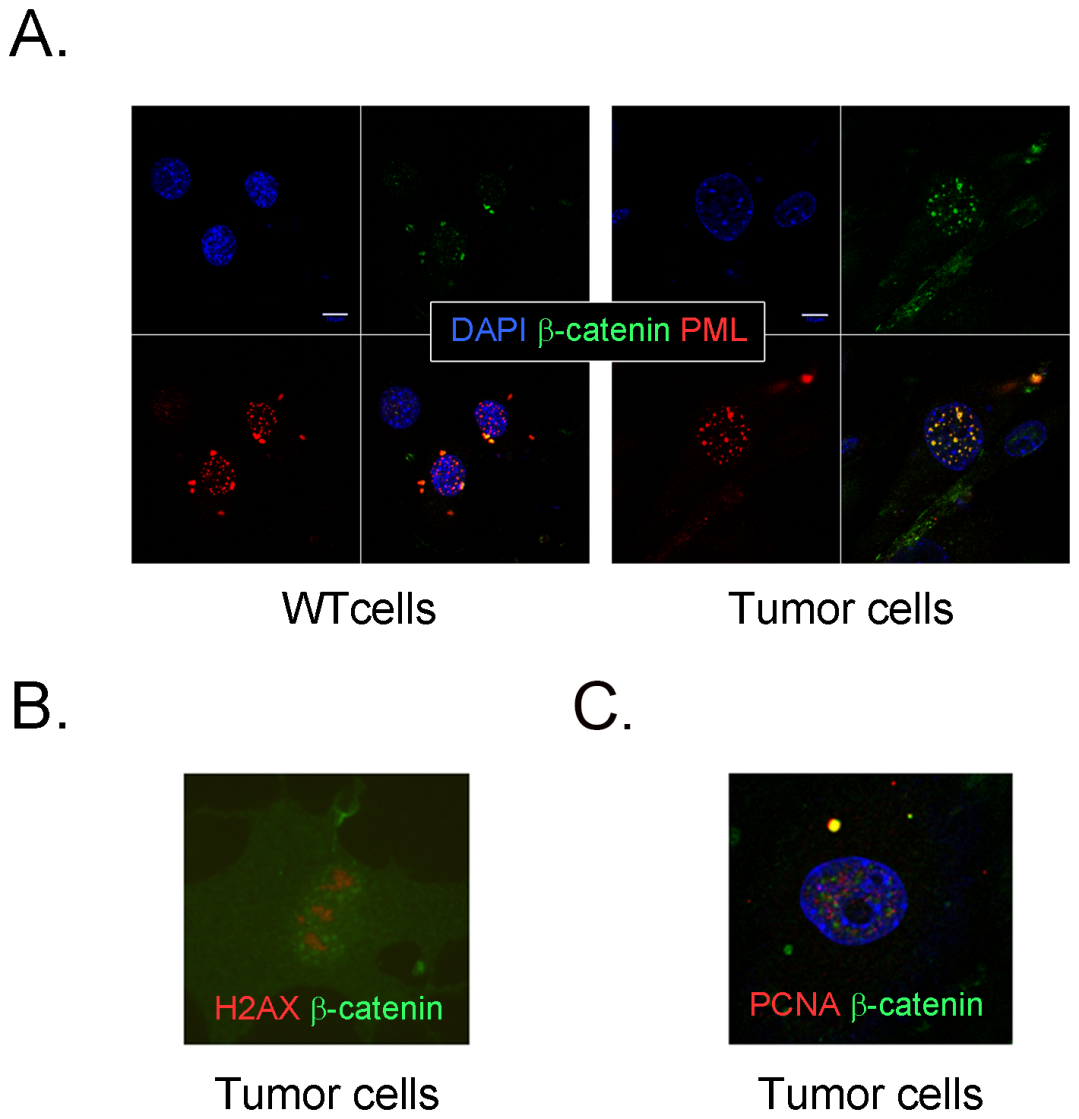
β-catenin (green) localizes to PML bodies in *Prkar1a^+/−^* tumor osteoblasts. A. Triple immunofluorescence for DAPI (blue), β-catenin (green), and PML (red) in WT or Tumor osteoblasts. The bottom right image in each panel represents the merged image. Scale bar: 10 µm. B. Immunofluorescence for H2AX and β-catenin in tumor cells. C. Immunofluorescence for PCNA and β-catenin. Note the lack of co-localization between proteins in B and C. Magnification (B and C): 630x.

In *Prkar1a^+/−^* tumor cells, IF analysis demonstrated that β-catenin exhibited strong co-localization with PML in tumor cells (Fig 2). Similar experiments with H2AX (either with or without prior cellular irradiation) or PCNA did not demonstrate co-localization of the fluorescent signals. Thus, we conclude that PKA activation is responsible for β-catenin relocalizing to PML bodies in the nuclei of tumor osteoblasts.

### MC3T3-E1 cells are a good model in which to study b-catenin relocalization

Because bone tumors from *Prkar1a^+/−^* mice bone tumors were heterogeneous, including elements of myxomatous, cartilaginous, and bony differentiation with aberrant bone architecture [4], we turned to the well-established MC3T3-E1 cell line to study the crosstalk between PKA and β-catenin in osteoblast cells. MC3T3-E1 cells are equivalent to committed preosteoblasts, which will lay down bone matrix and express markers of fully differentiated osteoblasts under differentiation conditions [30]. We had also previously generated MC3T3-E1 cells containing shRNA-mediated knockdown of *Prkar1a*, and showed that those cells had reduced differentiation [10].

**Figure 3:**
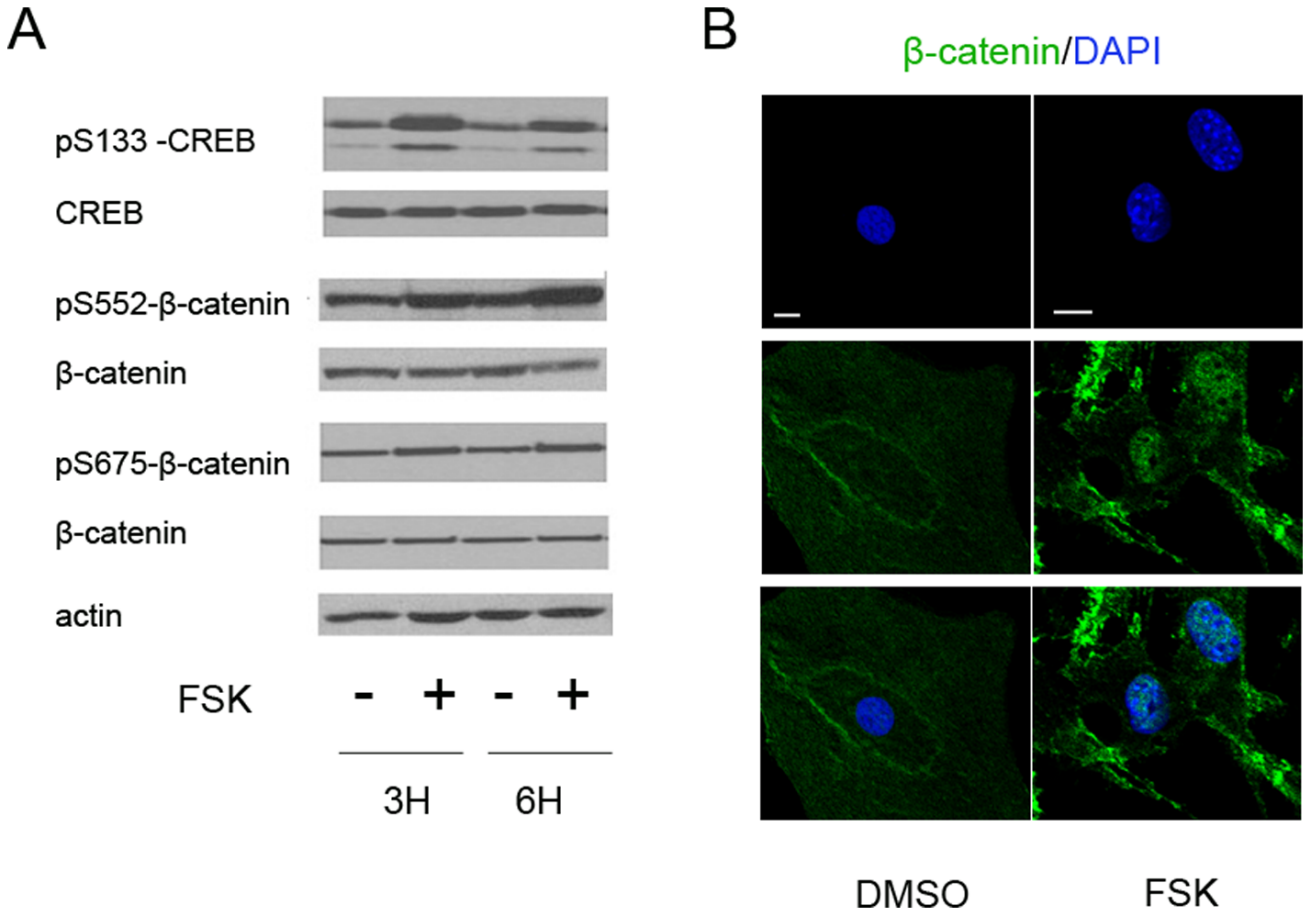
Stimulation of PKA by FSK increases β-catenin phosphorylation and nuclear relocalization in MC3T3-E1 cells. A. Protein lysates were prepared from MC3T3-E1 cells treated with vehicle for Forskolin (FSK) for the times indicated and blotted with the antibodies shown. Note the increases in pS133-CREB, pS552- and pS675- β-catenin at both timepoints without changes in total protein levels. Actin is shown as a loading control. 20 µg of protein were loaded per lane. B. Immunofluorescence tracking of β-catenin after vehicle (DMSO) or FSK treatment in MC3T3-E1 cells. The bottom row shows a merged images from the panels above. Scale bar: 10 µm.

As an initial step, we first sought to verify that β-catenin underwent PKA-mediated cellular redistribution in these cells. Cells were treated with forskolin (FSK, 50 µM) for 3 or 6 hours. As shown in Fig 3A, treatment of cells with FSK induced strong phosphorylation of the PKA target site Ser133 of the cAMP response element binding protein (CREB) at either timepoint. At the same time, enhanced phosphorylation of β-catenin at the S552 and S675 PKA sites was also observed. In accordance with the results in primary cultures from bone tumors, total β-catenin protein level was not changed in MC3T3-E1 cells after acute activation of PKA by forskolin treatment. Because treatment with forskolin for 3 hours and 6 hours generated similar results, we used 6 hour treatment with 50 µM for the remainder of our experiments.

To verify that β-catenin also underwent relocalization in response to PKA signaling in this model, we analyzed its subcellular localization after treatment as above. As Fig 3B demonstrates, untreated MC3T3-E1 cells demonstrate primarily β-catenin staining at the cell membranes, where it functions as a cytoskeletal adaptor protein. However, treatment of MC3T3-E1 cells with forskolin led to the movement of β-catenin into the cell nucleus, although the staining pattern was more diffuse than in the tumor cells.

### Role of PKA phosphorylation sites in intranuclear relocalization

**Figure 4:**
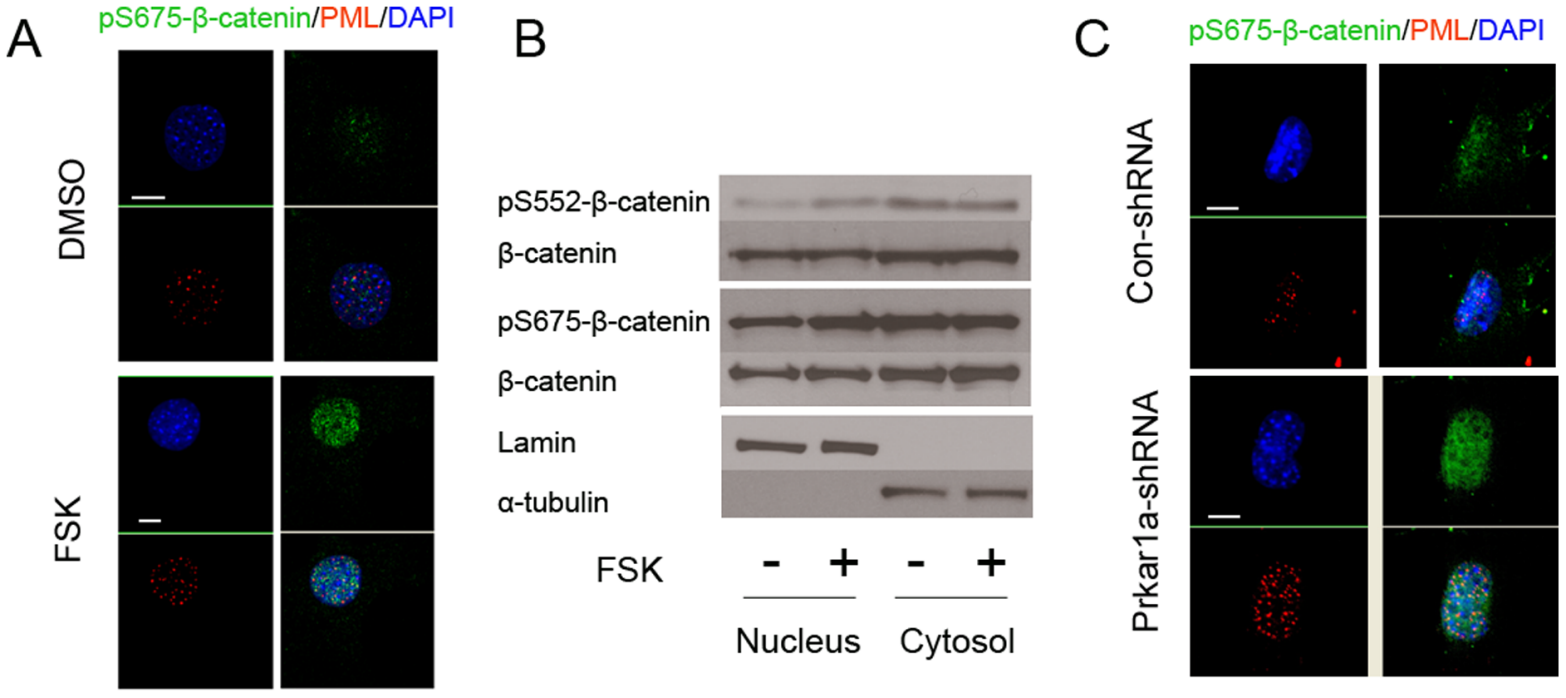
PKA activation promotes nuclear relocalization of phospho-β-catenin. A. Immunofluorescence for pS675-β-catenin (green), PML (red), with DAPI nuclear staining (blue) in MC3T3-E1 cells treated with vehicle (DMSO) or FSK. Note the nuclear accumulation of phospho-β-catenin in response to FSK. B. MC3T3-E1 cells were treated with vehicle or FSK and nuclear and cytosolic protein fractions prepared and blotted for the proteins shown. Specificity of the fractionation is demonstrated by blotting for Lamin A (nuclear marker) and α-tubulin (cytosolic marker). Note the enhanced phospho-β-catenin only in the nuclear fraction in response to FSK. 8 µg of nuclear and 20 µg of cytosolic protein were loaded per lane. C. Control or Prkar1a-knockdown MC3T3-E1 cells were studied by IF as in panel A. Scale bar for all images: 10 µm.

In order to better characterize the role of the PKA phosphorylation events in β-catenin's localization, we took advantage of phospho-specific antibodies that can detect the S675 and S552 phosphorylation events. Using immunofluorescence/confocal microscopy, we examined the subcellular distribution of pS675-β-catenin in response to FSK treatment. We observed that untreated cells had low levels of pSer675-β-catenin, but that the amount significantly increased after FSK; furthermore, the phosphoprotein was observed almost exclusively in the nucleus (Fig 4A). Efforts to repeat this analysis for pS552-β-catenin were unsuccessful, due to the fact that this antibody did not prove suitable for immunofluorescence.

Consistently, western blot analysis after cell fractionation detected an increase in both pS675- and pS552- β-catenin in the nuclei of cells treated with forskolin. Concurrent assessment of cytoplasmic levels of phospho-β-catenin demonstrated no change (Fig 4B). To ensure that nuclear exacts were not contaminated by cytosolic proteins or vice versa, nuclear and cytosolic extracts were also analyzed for the presence of the nuclear protein lamin A and the cytoplasmic protein α tubulin. These findings connected β-catenin signaling to PKA signaling in MC3T3-E1 cells and indicated a PKA-induced increase in phosphorylated β-catenin in the nucleus. Furthermore, treatment of the cells with the nuclear export inhibitor leptomycin B did not alter subcellular distribution of β-catenin (Figure S1), suggesting that nuclear export is not the limiting factor in the determination of its subcellular distribution.

To test whether the results obtained in forskolin treated cells could be reproduced by other means of PKA activation, we also utilized MC3T3-E1 cells carrying a siRNA-mediated knockdown (KD) of *Prkar1a*. These cells, which we have previously shown to have elevated PKA activity [10], also demonstrated a marked increase in nuclear pS675- β-catenin. Although there appeared to be a general increase in intranuclear β-catenin, there was overrepresentation of protein which co-localized in confocal analysis with PML bodies (Fig 4C). Thus, very similar results attained by two different approaches suggest that increased PKA activity promotes the association of β-catenin with PML in osteoblastic cells.

**Figure 5:**
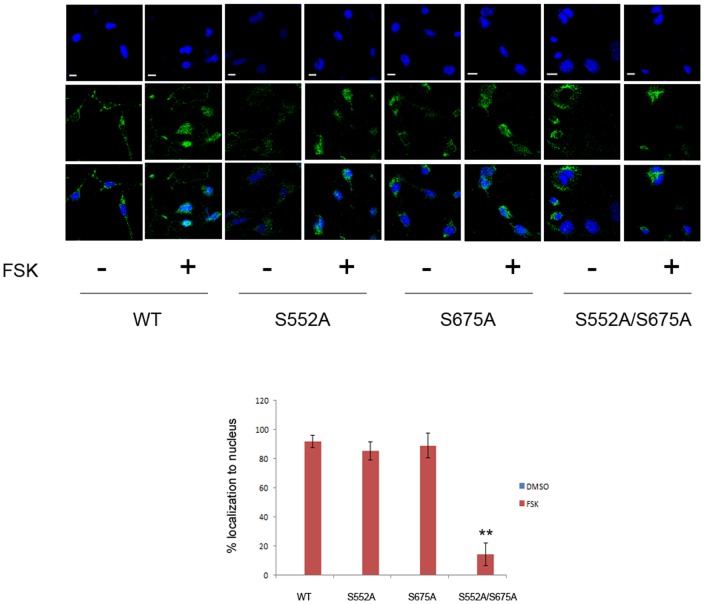
Mutation of phosphorylation sites affects nuclear localization of β-catenin. MC3T3-E1 cells were transfected with WT or mutated FLAG-tagged β-catenin constructs, treated with vehicle or forskolin for 6 h and then subjected to confocal microscopy with anti-FLAG antibodies. The bar graph shows the quantification of immunofluorescence data. 30-40 fields were counted for each transfection, yielding 70–100 transfected cells for each condition. Note that very little nuclear localization (<3%) occurs in the absence of FSK with any of the constructs used. Scale bars: 10 µm.

To determine if PKA phosphorylation sites within β-catenin are required for its nuclear translocation, we introduced mutations at S552 and/or S675 into FLAG-tagged β-catenin. We generated serine-to-alanine (S552A, S675A, and S552A/S675A) mutants to model non-phosphorylatable β-catenin, as well as serine-aspartate (S552D, S675D, and S552D/S675D) mutants to mimic the phosphorylated isoforms. Use of S>A and S>D mutants produced similar results, suggesting that the S>D mutants did not mimic the inducible phosphorylation of the protein. A similar phenomenon has been observed in the transcription factor CREB, where the S133D mutant behaves similarly to the S133A mutant [31] and in other PKA targets as well[32]. Because we observed that the S>A and S>D mutants behave similarly, we focused only on the S>A mutants. We transfected MC3T3-E1 cells with WT or mutant β-catenin and then treated cells with forskolin. As shown in Figure 5, WT or mutant β-catenin was primarily cytoplasmic in the absence of forskolin. After treatment with forskolin, single mutation of S552 or S675 to alanine residues had minimal effects on nuclear translocation; nevertheless, mutation of both sites profoundly reduced the nuclear accumulation of β-catenin. This data suggested that phosphorylation of β-catenin may induce a conformational change which cannot be mimicked by acidic residues and this structural alteration may be necessary for its nuclear translocation.

As confirmation, we obtained YFP-tagged β-catenin and introduced the same phosphosite mutants into this construct. Cells were co-transfected with CFP-tagged PML and co-localization were analyzed after treatment with DMSO or with FSK. Behavior of the fluorescently tagged proteins recapitulated that observed for the native proteins (Figure S1) confirming both that 1) S>A and S>D mutants exhibited the same behavior and 2) the PKA phosphorylation sites at S552 and S675 are necessary for its localization to PML bodies.

### PKA activation promotes TCF/LEF-dependent gene transcription

**Figure 6:**
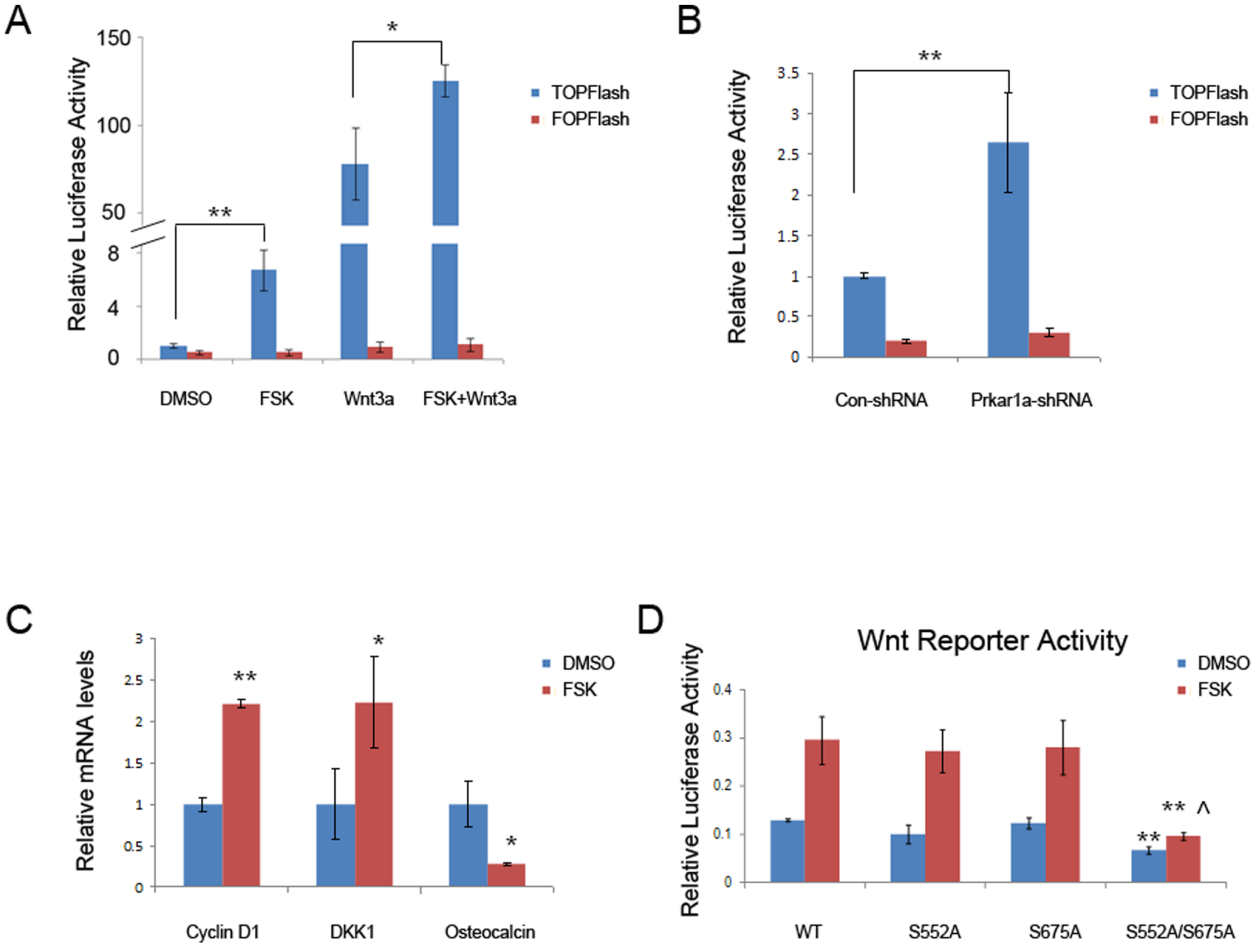
PKA activation enhances basal and stimulated Wnt/β-catenin -dependent transcriptional activity in MC3T3-E1 cells. A. Cells were transfected with a Wnt/β-catenin -reporter plasmid (TOPFlash) or the same plasmid with a mutation in the Wnt-responsive elements (FOPFlash). Luciferase assay was performed following treatment with vehicle, FSK, or Wnt3a (100 ng/ml) as indicated (* P<0.05, ** P<0.01). B. Luciferase assay was performed in cells with control or *Prkar1a* knockdown to measure the Wnt/β-catenin-reporter activity (** P<0.01). C. mRNA expression of Wnt/β-catenin target genes was determined using QPCR analysis. The expression of each was normalized to GAPDH expression (** P<0.01 versus DMSO treated cells). (D) Cells were transfected with TOPFlash or FOPFlash along with FLAG-tagged β-catenin or its PKA phosphorylation mutants, as indicated. The Wnt/β-catenin -reporter activity was measured by luciferase assay following stimulation of cells with forskolin. (** P<0.01 versus WT or single mutants, ∧ P<0.05 versus DMSO treated counterparts).

To understand the functional significance of PKA-dependent relocalization of β-catenin to PML bodies, we first examined how PKA activation affects β-catenin transcriptional activity. We found that forskolin stimulated a significant induction of the Wnt/β-catenin-dependent reporter TOPFlash without altering the control reporter FOPFlash in MC3T3-E1 cells. When cells were stimulated with the canonical Wnt ligand Wnt3a, levels of transcription increased approximately 75-fold, and PKA activation was able to provide an additional increase in transcriptional activity (Figure 6A). To rule out a non-specific effect of forskolin on Wnt/β-catenin-dependent promoter activity, we also used MC3T3-E1 cells with *Prkar1a* knockdown [13]. As shown in Figure 6B, the *Prkar1a* knockdown cells, which have increased PKA activity, showed higher TOPFlash reporter activity compared to the control cells.

We next examined whether PKA activation influences the expression of endogenous Wnt/β-catenin target genes by QPCR. Expression levels of *Ccnd1 (Cyclin D1)* and *Dkk1* (Dickkopf 1) were increased, whereas the mRNA level for *Osteocalcin* was decreased in cells treated with forskolin (Figure 6C). These findings are in accord with previous data reporting that *Cyclin D1* and *Dkk1* are positively regulated and *Osteocalcin* is negatively regulated by the Wnt/β-catenin pathway [12], [33], [34]. Collectively, these observations indicate that PKA activation, including pharmacological means or through reduction of *Prkar1a*, was responsible for the enhanced β-catenin-mediated transcription in osteoblastic cells, an observation which confirms previous data in other systems [19], [20], [35].

To study the role of the PKA phosphorylation sites in the transcriptional activation of β-catenin by PKA, we transfected MC3T3-E1 cells with WT or mutant β-catenin and measured activity of the TOPFlash reporter with or without FSK stimulation. As predicted from our above data, introduction of WT β-catenin and treatment with FSK showed about a 2.5-fold enhancement of reporter activity compared to the untreated cells. There was no activity in the assay when the FOPFlash reporter was used (data not shown). Use of the β-catenin mutants S552A or S675A showed similar effects, with both basal and FSK-stimulated levels not significantly different from the WT construct. However, mutation of both sites significantly decreased basal (unstimulated) activity, and also reduced the activity of the reporter after FSK induction. Although there was still a statistically significant increase in luciferase activity (p = 0.0.013), the induction was only ∼1.4-fold. Taken together, these results indicated that PKA promotes β-catenin-dependent transcription, at least in part, through phosphorylation of β-catenin at S552 or S675.

**Figure 7:**
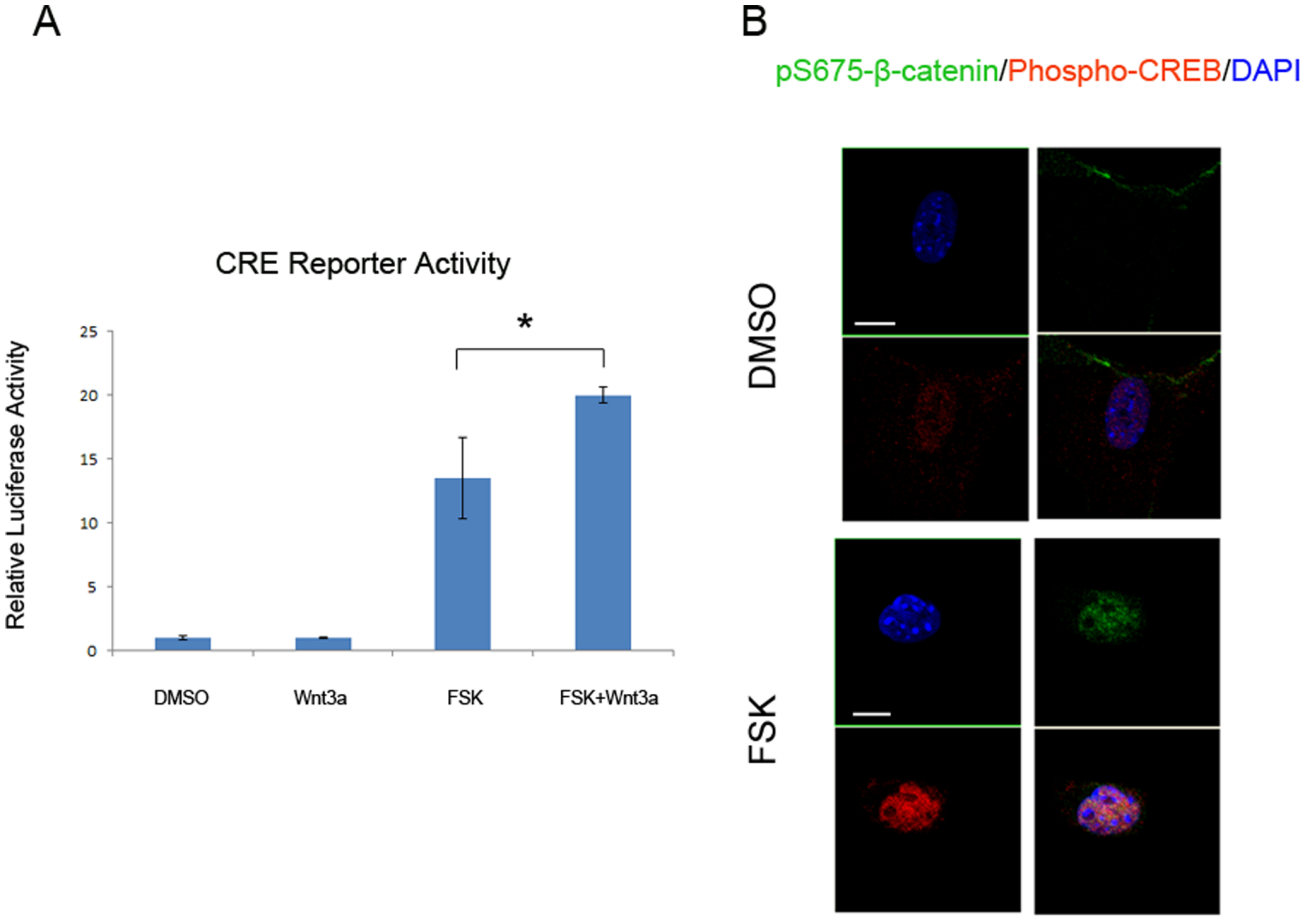
CREB and β-catenin cooperation promotes cAMP-dependent transcription. A. MC3T3-E1 cells were transfected with a cAMP responsive element luciferase construct. Luciferase assay was performed following stimulation of vehicle, FSK, or Wnt3a (100 ng/ml) as indicated (* P<0.05). Error bars represent standard deviation. B. IF co-localization of pS675β-catenin (green) and pS133-CREB (red) in response to FSK stimulation. DAPI (blue) stains the nucleus. The bottom right panel of each group shows the merged image. Scale bar: 10 µm.

To test if PKA-Wnt crosstalk flows in both directions, we transfected MC3T3-E1 cells with a cAMP responsive element-luciferase construct and stimulated the cells with vehicle, forskolin or Wnt3a. As shown in Figure 7A, Wnt3a induced more PKA-dependent transcription, indicating synergic actions of forskolin and Wnt3a on β-catenin-dependent as well as PKA-dependent transactivation. The cooperation of PKA and Wnt/β-catenin signaling was further confirmed by nuclear co-localization of phosphorylated (activated) pS133-CREB and pS675-β-catenin (Fig 7B).

### CREB and β-catenin co-regulate gene transcription

To assess the functional interaction of PKA and β-catenin targets, we turned to data we had previously generated on mRNA transcripts altered in *Prkar1a^+/−^* bone tumors [23]. In that analysis, we identified 140 known genes which exhibited altered transcription in tumor cells compared to WT osteoblasts, including 57 genes upregulated and 83 genes downregulated (Full dataset included as Table S1).

To predict potential interactions between CREB and TCF/β-catenin in co-regulating gene transcription, we analyzed the promoters of this gene set for CREB or TCF/β-catenin binding sites. For this analysis, the promoter sequences (−5000 bp upstream to +2000 bp downstream of known Transcription Start Site) were retrieved from the MPromDb database [36]. These sequences were scanned with the program MATCH in search of CREB and TCF binding sites, using the position weight matrices from TRANSFAC database (TRANSFAC 9.1, minSum profile) [37]. From this analysis (Table 1), we determined that 65% of promoters had binding sites for both factors, whereas only 21.4% had sites for neither. Of the remainder, 6.4% had a predict ted Tcf site only, where as 7.1% had only a CRE site. The percentage of genes with both sites that were upregulated or downregulated were similar for those genes that had both sites (59.6% upregulated vs. 68.7% downregulated) or had neither site (21.1% upregulated vs. 21.7% downregulated). Interestingly, of the upregulated genes, 17.5% had only a CRE site, whereas only 1.8% only had a Tcf site (p<−0.0001 by Fisher's exact test). Conversely, genes that were downregulated were much more likely to have only a Tcf site (9.6%) than only a CRE site.(0%) (Table 1)

**Table 1:** Distribution of TCF and CREB binding sites in genes with altered transcription in *Prkar1a^+/−^ bone tumors* *.

	Neither (%)	CREB only (%)	TCF only (%)	Both (%)
**All genes (N = 140)**	**28 (20%)**	**10 (7.1%)**	**9 (6.4%)**	**93 (66.4%)**
**Upregulated genes (n = 57)**	**12 (21.1)**	**10 (17.5)**	**1 (1.8)**	**34 (59.6)**
**Downregulated genes (N = 83)**	**16 (19.3)**	**0 (0)**	**8 (9.6)**	**59 (71.1)**

*Distribution of promoters with neither or both sites is NS. Distribution of Tcf sites vs. up- and down-regulated genes has p = 0.037 by Fisher's exact test. Distribution of CREB and TCF sites vs. up- and down-regulated genes shows p<0.0001 by Fisher's exact test.

### PKA activation represses Wnt5a/Ror2 non-canonical Wnt pathway

**Figure 8:**
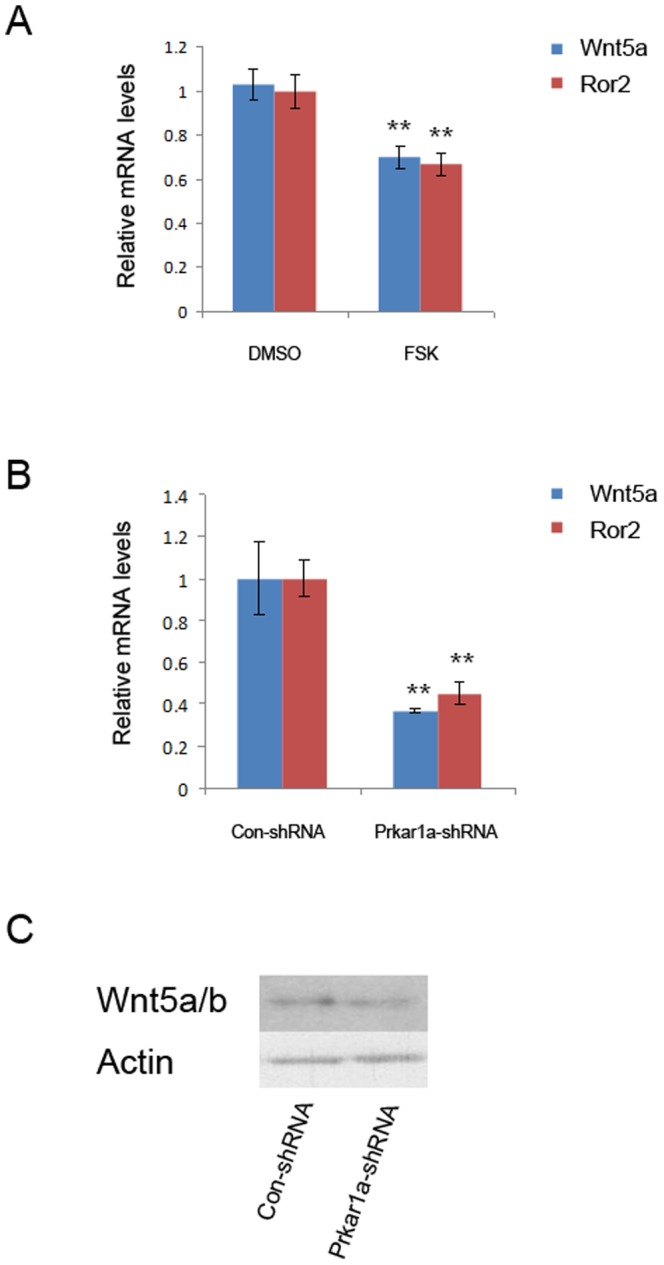
PKA activation represses Wnt5a/Ror2 pathway. A. and B. mRNA expression of Wnt5a and Ror2 was determined using QPCR analysis in MC3T3-E1 cells treated with FSK (A) or with *Prkar1a* knockdown (B) (** P<0.01 versus DMSO or control shRNA treated cells). Error bars represent standard deviation. C. 20 ug of protein lysates from MC3T3-E1 cells were analyzed for Wnt5a/b by Western blotting. Actin was used as the internal control. This experiment was repeated at least twice with similar results, and a representative blot is shown.

Because it has been shown that non-canonical Wnt signaling mediated by Wnt5a/Ror2 interactions can inhibit canonical Wnt/β-catenin signaling at the level of TCF-mediated transcription [38], we sought to examine whether PKA activation affects this pathway in MC3T3-E1 cells. As shown in Figure 8, both mRNA and protein levels of Wnt5a were decreased in MC3T3-E1 cells treated with forskolin or with *Prkar1a* knockdown; and Ror2 mRNA expression was also reduced in cells treated with forskolin or with *Prkar1a* knockdown. Therefore, these data indicate that suppression of Wnt5a/Ror2 pathway occurs concurrently with activation of the Wnt/β-catenin signaling caused by PKA activation.

## Discussion

Crosstalk between the PKA and Wnt/β-catenin signaling pathways has been known since the mid 2000's, when it was reported that β-catenin was a substrate for PKA [19], [20]. Most reports have indicated that PKA activation can stimulate β-catenin transcriptional activity, although the mechanism of this effect may differ depending on the cellular context [19], [20]. Conversely, inhibition of PKA activity either upstream [39] or at the kinase itself [40] has been shown to reduce β-catenin activity. In this report, we investigate this same phenomenon in cells of the osteoblast lineage, either primary bone tumors from *Prakr1a^+/−^* mice or the well-established MC3T3-E1 cell line. Our data indicates that in this system, β-catenin activation occurs without an overall increase in levels of this protein. However, dissection of the mechanism by which this occurred revealed the striking finding that β-catenin undergoes PKA- dependent relocalization to nuclear PML bodies. We propose that localization at this intranuclear structure enables β-catenin to interact with other transcriptional components (including phosphoCREB) and enables enhanced β-catenin-dependent transcription.

PML is a multifunctional protein with multiple splice isoforms that play roles in a variety of intranuclear functions, including DNA damage response, apoptosis, senescence, and transcriptional activation. [26]. In the present analysis, we propose that PML is activating a site for the assembly of transcriptional complexes that contain β-catenin. It has previously been reported that Tcf4 and β-catenin can bind to PML in colorectal cancer cell lines [41], although that report indicated that only PML isoform IV was bound to β-catenin. In the present work, we have not dissected which PML isoform was involved in the observed binding, but this binding was observed both with native PML and with an expression construct corresponding to PML isoform III [25].

One of the roles of PML is to affect gene transcription, and the studies described here indicate that the ability to modulate transcription of the luciferase reporter construct is strongly correlated with the ability to bind PML in the nucleus. Unlike prior studies, we found that mutation of one of the PKA sites within β-catenin was insufficient to suppress its transcriptional activity or its entry into a PML complex. However, when both PKA sites (S552 and S675) were mutated, both abilities were lost.

In terms of a mechanism, the most likely explanation for these observations is that PKA-mediated phosphorylation of β-catenin promotes its transport into the nucleus, where it is then available to bind to PML and PML-associated complexes. PKA also may potentiate intranuclear β-catenin by interfering with the ability of nuclear APC to chaperone it out of the nucleus [42]. However, when both PKA sites are mutated, β-catenin does not undergo nuclear import. This analysis is supported by the fact of two observations. First, S552A/S675A-β-catenin –YFP fusions proteins rarely get into the nucleus; however, when found in the nucleus they co-localize with PML (Figure S2). Second, co-IP experiments (Figure S3) indicate that both WT and S552A/S675A-β-catenin are able to pull down PML in whole cell lysates. We would propose that *in situ* crosslinking experiments would not identify a complex between and S552A/S675A-β-catenin and PML because these proteins are not localized within the same cellular compartment. These experiments, however, have not been performed.

Alternative explanations might include β-catenin binding to other proteins in the PML complexes, and that these collaborative binding interactions are disrupted by the S>A mutations.

Analysis of the genes altered in bone tumors from *Prkar1a^+/−^* mice also suggest the cooperation of the cAMP-responsive transcription factor CREB and β-catenin, as binding sites for CREB and TCF strongly tended to occur together in the promoters of genes with altered transcription in the tumors. As TCF sites were more commonly observed in isolation in gene promoters whose transcripts were reduced in tumors, we propose that TCF/β-catenin primarily acts as a transcription suppressor in osteoblasts, whereas CREB primarily promotes gene transcription. However, when both TCF and CREB sites are found (which presumably happens in the context of a PML body), transcriptional effects are much more variable. Interestingly, one of the actions of recruitment of β-catenin to PML bodies appears to be a suppression of non-canonical Wnt signaling including Wnt5a and Ror2. Although we have not formally investigated whether suppression of Wnt/Ror2 signaling causes enhancement of the Wnt/β-catenin pathway, this association has been reported in other systems [43], [44] and may provide a secondary means by which the canonical Wnt/β-catenin pathway is activated in our cells.

Overall, we have used a combined *in vivo* and *in vitro* approach to study the interaction of PKA with Wnt/β-catenin signaling. We make the surprising observation that PKA drives β-catenin into the nucleus, where it binds to PML bodies to exert transcriptional effects on Wnt/β-catenin target genes. There is also secondary inhibition of competing non-canonical Wnt/Ror2 signaling. Although some of our data confirms prior studies, the fact that this data was generated in a more physiologic system in a tissue type where both PKA and Wnt signaling are known to play significant roles has enabled us to shed new light on intranuclear β-catenin function and add important information to the understanding of the modes of crosstalk between these central signaling cascades.

## Supporting Information

Figure S1 **Leptomycin does not altered β-catenin localization.** WT osteoblasts were treated with the nuclear export inhibitor Leptomycin B with or without Forskolin (FSK) and probed for β-catenin localization by immunofluorescence. Nuclei are counterstained with DAPI. Note that FSK induced nuclear accumulation of β-catenin but that leptomycin B did not produce further alterations. Scale bar: 10 µm.
(TIF)

Figure S2 **PKA activation promotes β-catenin to co-localize with PML bodies.** A. Primary osteoblasts were co-transfected with WT or mutant YFP-tagged β-catenin and CFP-PML and treated with forskolin. Proteins were visualized by immunofluorescence confocal microscopy and counted to determine nuclear localization of β-catenin as well as co-localization with PML. B. Quantitation of data on transfected cells. At least 100 transfected cells were counted in each assay. Note that the S>A and S>D mutants behave in the same fashion, and that these results closely parallel those shown in Figure 5. Scale bar: 10 µm
(TIF)

Figure S3 **β-catenin co-immunoprecipiates with PML.** A. MC3T3-E1 pre-osteoblasts were treated with Forkolin (Fsk) or vehicle (Ctl) and subject to immuneprecipitation (IP) with primary antibodies against β-catenin or PML. After SDS-PAGE, the blots were probed with anti-β-catenin or anti-PML, as indicated. The arrows at the left of each panel indicate the respective proteins. Note that Fsk does not alter the interaction between β-catenin (94 kDa) and PML (78/97 kDa). The vertical white line in the right panel indicates that different exposure times for this blot are shown for the two images. B. MC3T3-E1 cells were transfected with CFP-PML and Flag-tagged β-catenin (WT or S552A/S675A double mutant) and treated with vehicle (Ctl) or Fsk. Lysates were immunoprecipitated with anti-Flag or anti-CFP and immunoblotted with the other antibody as indicated. As in panel (A), no differences were detected irrespective of the use of the β-catenin mutants or Fsk treatment.
(TIF)

Table S1 **Primer sequences for real-time PCR of mouse RNA targets.**
(DOCX)
