# Supplementary figures and images for: Protein Kinase A Activation Enhances β-Catenin Transcriptional Activity through Nuclear Localization to PML Bodies
Source: PLoS One. 2014 Oct 9;9(10):e109523. doi: 10.1371/journal.pone.0109523 (PMC4192022; doi:10.1371/journal.pone.0109523)

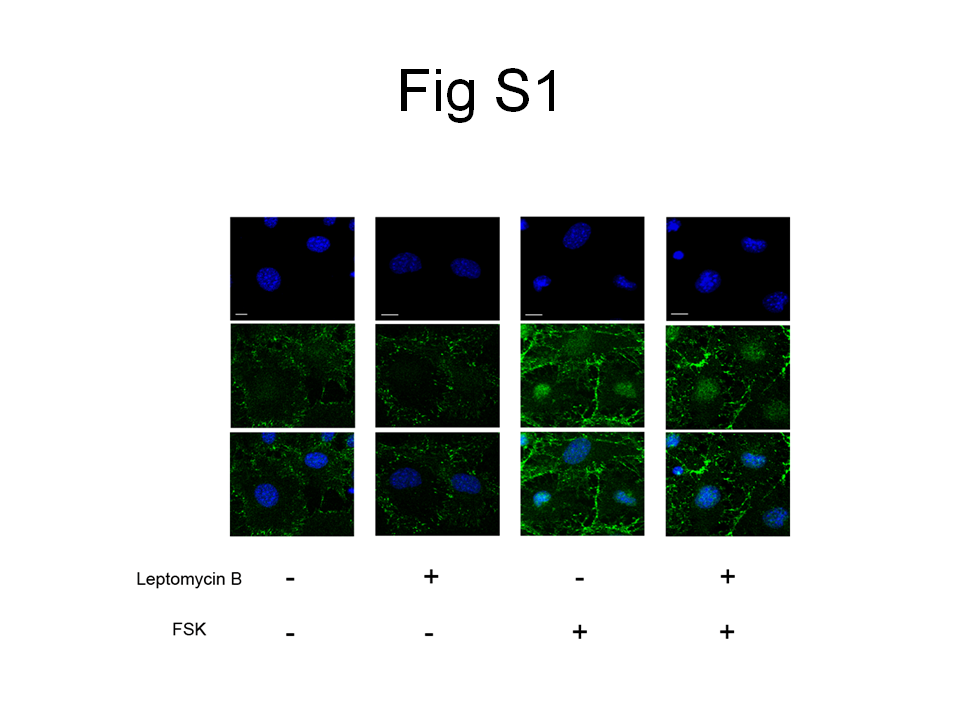

Supplement: Figure S1 — Leptomycin does not altered β-catenin localization. WT osteoblasts were treated with the nuclear export inhibitor Leptomycin B with or without Forskolin (FSK) and probed for β-catenin localization by immunofluorescence. Nuclei are counterstained with DAPI. Note that FSK induced nuclear accumulation of β-catenin but that leptomycin B did not produce further alterations. Scale bar: 10 µm. (TIF) [file pone.0109523.s001.tif]

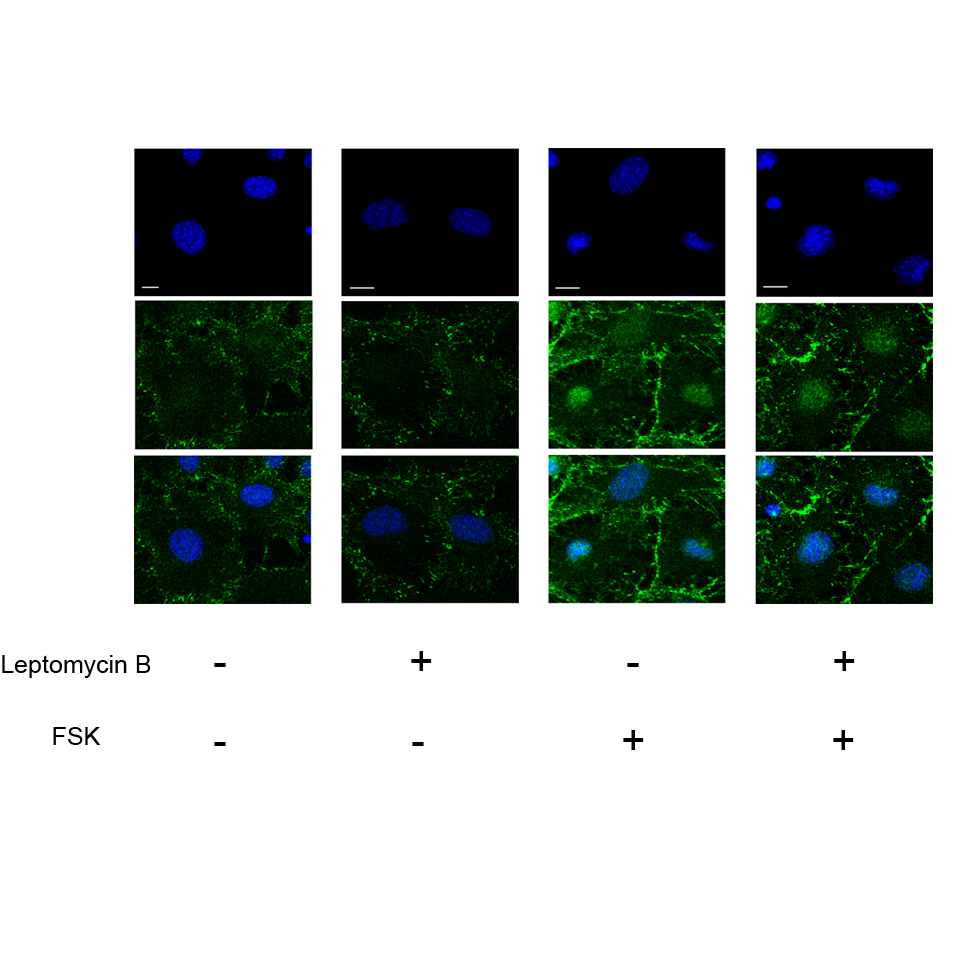

Supplement: Figure S2 — PKA activation promotes β-catenin to co-localize with PML bodies. A. Primary osteoblasts were co-transfected with WT or mutant YFP-tagged β-catenin and CFP-PML and treated with forskolin. Proteins were visualized by immunofluorescence confocal microscopy and counted to determine nuclear localization of β-catenin as well as co-localization with PML. B. Quantitation of data on transfected cells. At least 100 transfected cells were counted in each assay. Note that the S>A and S>D mutants behave in the same fashion, and that these results closely parallel those shown in Figure 5. Scale bar: 10 µm (TIF) [file pone.0109523.s002.tif]

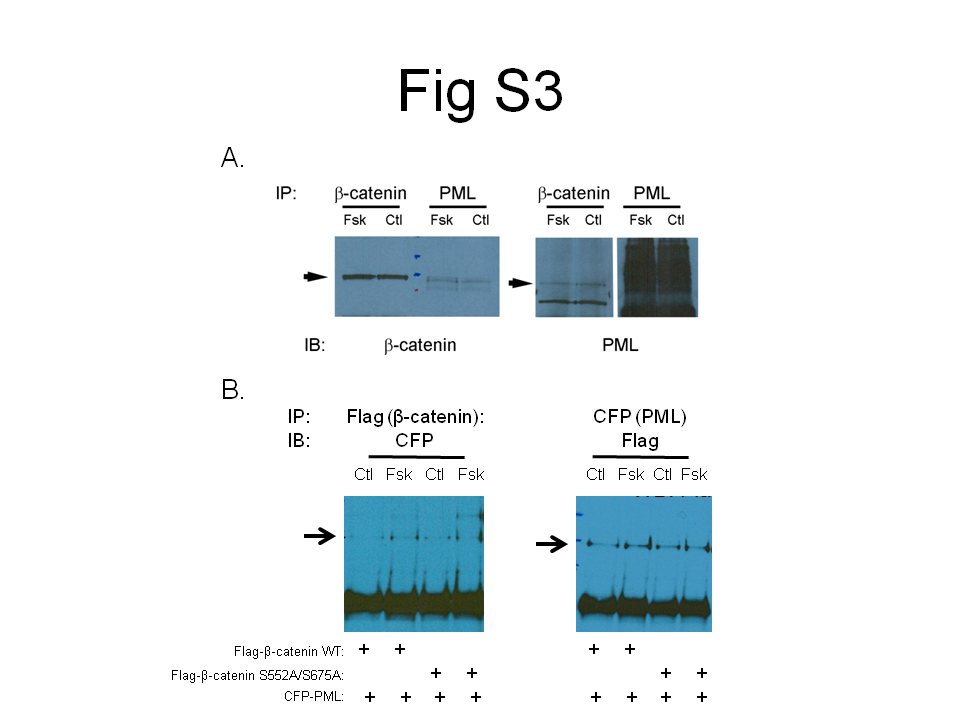

Supplement: Figure S3 — β-catenin co-immunoprecipiates with PML. A. MC3T3-E1 pre-osteoblasts were treated with Forkolin (Fsk) or vehicle (Ctl) and subject to immuneprecipitation (IP) with primary antibodies against β-catenin or PML. After SDS-PAGE, the blots were probed with anti-β-catenin or anti-PML, as indicated. The arrows at the left of each panel indicate the respective proteins. Note that Fsk does not alter the interaction between β-catenin (94 kDa) and PML (78/97 kDa). The vertical white line in the right panel indicates that different exposure times for this blot are shown for the two images. B. MC3T3-E1 cells were transfected with CFP-PML and Flag-tagged β-catenin (WT or S552A/S675A double mutant) and treated with vehicle (Ctl) or Fsk. Lysates were immunoprecipitated with anti-Flag or anti-CFP and immunoblotted with the other antibody as indicated. As in panel (A), no differences were detected irrespective of the use of the β-catenin mutants or Fsk treatment. (TIF) [file pone.0109523.s003.tif]
