# Supplementary material for: Protein Kinase A Activation Enhances β-Catenin Transcriptional Activity through Nuclear Localization to PML Bodies
Source: PLoS One. 2014 Oct 9;9(10):e109523. doi: 10.1371/journal.pone.0109523 (PMC4192022; doi:10.1371/journal.pone.0109523)

Supplemental Table1 Primer sequences for real-time PCR of mouse RNA targets

| Transcript | Left | Right |
| --- | --- | --- |
| Cyclin D1 | ATGTGAAGTTCATTTCCAACCC | TTGACTCCAGAAGGGCTTCA |
| DKK1 | GGAGGTCCCGAAGTTGAGGT | CGCAAGGGTAGGGCTGGTAG |
| Osteocalcin | AGGACCATCTTTCTGCTCACT | TTCACTACCTTATTGCCCTCC |
| Wnt5a | AGGAGTTCGTGGACGCTAGA | GCCGCGCTATCATACTTCTC |
| Ror2 | CCCAACTTCTACCCAGTCCA | TGTCCGCCACAGATGTATTG |

 


Supplement: Table S1 — Primer sequences for real-time PCR of mouse RNA targets. (DOCX) [file pone.0109523.s004.docx]
